# The leader peptide of mutacin 1140 has distinct structural components compared to related class I lantibiotics

**DOI:** 10.1002/mbo3.222

**Published:** 2014-11-17

**Authors:** Jerome Escano, Byron Stauffer, Jacob Brennan, Monica Bullock, Leif Smith

**Affiliations:** Department of Biological Sciences, Texas A&M UniversityCollege Station, Texas, 77843

**Keywords:** Antibiotic biosynthesis, lantibiotic, leader peptide, mutacin 1140, secondary metabolite

## Abstract

Lantibiotics are ribosomally synthesized peptide antibiotics composed of an N-terminal leader peptide that promotes the core peptide's interaction with the post translational modification (PTM) enzymes. Following PTMs, mutacin 1140 is transported out of the cell and the leader peptide is cleaved to yield the antibacterial peptide. Mutacin 1140 leader peptide is structurally unique compared to other class I lantibiotic leader peptides. Herein, we further our understanding of the structural differences of mutacin 1140 leader peptide with regard to other class I leader peptides. We have determined that the length of the leader peptide is important for the biosynthesis of mutacin 1140. We have also determined that mutacin 1140 leader peptide contains a novel four amino acid motif compared to related lantibiotics. PTM enzyme recognition of the leader peptide appears to be evolutionarily distinct from related class I lantibiotics. Our study on mutacin 1140 leader peptide provides a basis for future studies aimed at understanding its interaction with the PTM enzymes.

## Introduction

**Figure 1:**
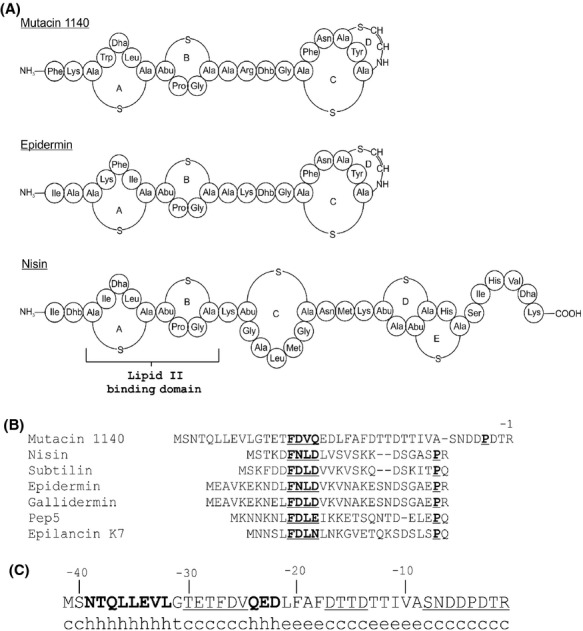
Lantibiotic structural elements. (A) Covalent structures for mutacin 1140, epidermin, and nisin with the lanthionine rings labeled from N- to C-terminus. (B) Leader sequence alignments of structurally related class I type AI lantibiotics; that is, mutacin 1140 produced by *Streptococcus mutans*, nisin produced by *Lactococcus lactis*, subtilin produced by *Bacillus subtilis*, epidermin produced by *Staphylococcus epidermidis*, gallidermin produced by *Staphylococcus gallinarium*, Pep5 produced by *Staphylococcus epidermidis,* epilancin K7 produced by *Staphylococcus epidermidis* (Gross and Morell 1971; Allgaier et al. 1986; Kellner et al. 1988; Kaletta et al. 1989; Piard et al. 1992; Klein and Entian 1994; Vandekamp et al. 1995; Hillman et al. 1998). (C) Secondary structure prediction using SOPMA for mutacin 1140 leader peptide; h (alpha helix), e (extended strand), c (random coil), t (beta turn). Alpha helical regions are in bold, while random coils are underlined in the leader peptide sequence.

Many strains of medically important bacteria have become increasingly resistant to currently available antibiotics. Healthcare-associated infections caused by multi-drug-resistant pathogens are leading to longer hospital stays and increased mortality. Worldwide, millions suffer from antibiotic-resistant infections, which results in a huge cost to the healthcare system. The development of new antibiotics has become a critical, unmet need in the medical community (Talbot et al. 2006; Laible 2014; Lushniak 2014). Lantibiotics are an important class of antibiotics with potential clinical relevance for the treatment of antibiotic-resistant Gram-positive bacteria (Chatterjee et al. 2005; Lubelski et al. 2008; Smith and Hillman 2008; Oman and van der Donk 2010). Lantibiotics acquired their name because of the characteristic lanthionine rings found in the bioactive core peptide. Lantibiotics also contain an array of unusual amino acids such as 2,3-didehydroalanine (Dha), 2, 3-didehydrobutyrine (Dhb), S-amino vinyl-D-cysteine (AviCys), aminobutyrate (Abu), 2-oxopropionyl, 2-oxobutyryl, and hydroxypropionyl (Chatterjee et al. 2005; Lubelski et al. 2008; Smith and Hillman 2008; Oman and van der Donk 2010). The molecular structure of mutacin 1140 contains four macrocyclic rings (see Fig. 1A), each of which contains a lanthionine or methyllanthionine residue. Mutacin 1140 also contains the post translationally modified amino acid residues 2,3-didehydroalanine (Dha), 2, 3-didehydrobutyrine (Dhb), and S-amino vinyl-D-cysteine (AviCys) (Smith et al. 2000, 2003). Mutacin 1140 rings A and B (see Fig. 1A), the lipid II-binding domain, is similar to the class I lantibiotics nisin and epidermin (Smith et al. 2000, 2003). It was discovered that both nisin and mutacin 1140 abduct lipid II from the site of new cell wall synthesis, ultimately causing cell death (Hasper et al. 2006; Smith et al. 2008).

Mutacin 1140 is synthesized by the Gram-positive oral bacterium *Streptococcus mutans* JH1140 (Hillman et al. 1998; Smith et al. 2000). Mutacin 1140 was shown to have low micromolar to submicromolar minimum inhibitory concentration (MIC) against several species of Gram-positive pathogens (Ghobrial et al. 2009). The study by Ghobrial et al. further demonstrated that mutacin 1140 is bactericidal against *Streptococcus pneumoniae* and multi-drug-resistant strains of *Staphylococcus aureus*, bacteriostatic against vancomycin-resistant *Enterococcus faecium* (VREF), and had no activity against Gram-negative bacteria or yeast. The study demonstrated that mutacin 1140's time-kill profile against select pathogens was similar to those of vancomycin, which also binds to lipid II (Breukink and de Kruijff 2006). Furthermore, mutacin 1140 had a low *in vitro* cytotoxicity, was well tolerated in murine models when administered intravenously, and was found to be distributed in all body compartments (Ghobrial et al. 2010). Demonstration of efficacy was achieved in a pilot study in which 60 times the LD_50_ of *S. aureus* was administered in a rat peritonitis model (Ghobrial et al. 2010). Development of significant resistance was not observed during repeated subculture of *S. aureus* or *S. pneumoniae* in medium containing sub-lethal concentrations of mutacin 1140 (Ghobrial et al. 2009). The basis for this observation may be due, in part, to the fact that the molecular target, the isoprene, pyrophosphate, and N-acetylmuramic acid of lipid II, is evolutionarily ancient and highly conserved throughout the bacterial kingdom, indicating that mutations within this structural element of lipid II may be prohibited. Based on these and other studies, mutacin 1140 has the potential to replace current, failing drugs of last resort and serve in the treatment of problematic infections caused by Gram-positive bacteria such as methicillin-resistant *S. aureus*, vancomycin-resistant Enterococci, and *Clostridium difficile*.

A better understanding of the biosynthesis of mutacin 1140 may promote the production and purification of mutacin 1140 or core peptide variants of mutacin 1140. Several core peptide variants of mutacin 1140 have been previously engineered to have enhanced bioactivity (Chen et al. 2013). The mutacin 1140 leader peptide sequence is distinct from the leader peptide of related class I lantibiotics (Fig. 1B). Class I leader peptides have conserved structural elements. The common motif found in class I lantibiotics is the F(N/D)LD box and a proline at the (-2) position. Several mutagenesis studies with the lantibiotic nisin biosynthesis system show that the box motif is important for the maturation of the core peptide antibiotic (Plat et al. 2011, 2013; Abts et al. 2013). Additional experimental studies suggest that the leader peptide is important for interacting with the dehydratase LanB, cyclase LanC, and transporter LanT(Chen et al. 2001; Kuipers et al. 2004; Furgerson Ihnken et al. 2008). However, these studies are challenged by the failure to distinguish between which of the proposed leader peptide functions are affected by the changes in amino acid residues.

In this study, we have identified structural regions in the leader peptide that are important for the biosynthesis of mutacin 1140 core peptide. We have determined that the leader peptide function is not dependent on the amino acid sequence except for a four amino acid region within the center of the leader peptide. These four amino acids comprise a novel four amino acid motif with the latter two amino acids being essential for leader peptide function. This motif is different in amino acid properties found in other class I lantibiotic motifs in related lantibiotic groups such as nisin and epidermin. Mutacin 1140 leader peptide is also longer than other class I lantibiotic leader peptides and we have determined that the additional length of the peptide of mutacin 1140 is important for efficient biosynthesis. These studies advance our understanding of the biosynthesis of mutacin 1140 and will promote future studies aimed at furthering our understanding of the leader peptide's interaction with the post translational modification (PTM) modification enzymes.

## Materials and Methods

### Bacterial strains, plasmids, and media

Bacterial strains and plasmids used in this study are listed in Table 1. The cloning strain *Escherichia coli* DH5*α* (Invitrogen, Carlsbad, CA) was cultured at 37°C on Luria-Bertani (LB) broth or agar. THyex broth (30 g/L Todd Hewitt Broth, 3 g/L yeast extract), THyex agar media (30 g/L Todd Hewitt Broth, 3 g/L yeast extract, 15 g/L agar; Bacto, Sparks, MD), and Top agar media (30 g/L Todd Hewitt Broth, 3 g/L yeast extract, 7.5 g/L agar; Bacto) were used to culture *S. mutans* JH1140 ATCC 55676 and *Micrococcus luteus* ATCC 10240 at 37°C.

**Table 1:** Strains and plasmids used in this study. All mutations were made in the wild-type strain JH1140. All the plasmids came from *Escherichia coli* DH5*α* cells

Strains used	Plasmid intermediate	Description	References
*S. mutans*			
*JH1140 ATCC 55676*		Wild-type bacteriocin-producing strain	Strain (Hillman et al. 1998)
*lanA*:IFDC2	pIFDC2	Gene replacement strain	Plasmid (Xie et al. 2011)
Δ(-40-33)	pΔ(-40-33)	8 amino acid(AA) N-terminal truncation	This study
Δ(-40-28)	pΔ(-40-28)	13 AA N-terminal truncation	This study
Δ(-40-24)	pΔ(-40-24)	17 AA N-terminal truncation	This study
Δ(-40-37)	pΔ(-40-37)	4 AA N-terminal truncation	This study
Δ(-36-33)	pΔ(-36-33)	Internal 4 AA truncation	This study
Δ(-32-28)	pΔ(-32-28)	Internal 4 AA truncation	This study
Δ(-27-24)	pΔ(-27-24)	Internal 4 AA truncation	This study
Δ(-23-20)	pΔ(-23-20)	Internal 4 AA truncation	This study
Δ(-7-2)*	pΔ(-7-2)	Internal 6 AA truncation	This study
F(-27)A	pF(-27)A	Single alanine substitution at presumed box	This study
D(-26)A	pD(-26)A	Single alanine substitution at presumed box	This study
V(-25)A	pV(-25)A	Single alanine substitution at presumed box	This study
Q(-24)A	pQ(-24)A	Single alanine substitution at presumed box	This study
Q(-24)InsAla	p Q(-24)InsAla)	Alanine insertion at EDLF motif	This study
E(-23)A	pE(-23)A	Single alanine substitution at EDLF motif	This study
E(-23)InsAla	p E(-23)InsAla	Alanine insertion at EDLF motif	This study
D(-22)A	pD(-22)A	Single alanine substitution at EDLF motif	This study
D(-22)InsAla	p D(-22)InsAla	Alanine insertion at EDLF motif	This study
L(-21)A	pL(-21)A	Single alanine substitution at EDLF motif	This study
Δ(-21)	pΔ(-21)	Single AA truncation at EDLF motif	This study
L(-21)InsAla	p L(-21)InsAla	Alanine insertion at EDLF motif	This study
F(-20)A	pF(-20)A	Single alanine substitution at EDLF motif	This study
Δ(-20)	pΔ(-20)	Single AA truncation at EDLF motif	This study
Δ(-19)	pΔ(-19)	Single AA truncation at EDLF motif	This study
AALF	pAALF	Double alanine substitution at ED	This study
EDED	pEDED	ED substitution for LF	This study
N+6xHis*	pN+6xhis	6xHis addition at N-terminus	This study
N6xHis*	pN6xhis	6xHis substitution at the N-terminus	This study
*M. luteus* ATCC 10760		Indicator strain in the differed antagonism assay	Strain (Chen et al. 2013)
*E. coli* DH5 *α*	pCR®2.1-TOPO®	Intermediate cloning host	Invitrogen®

### Mutagenesis of the leader peptide

The *S. mutans* genome database and *lan* gene cluster, GenBank/EMBL accession number (AF051560), was used to design primers for the mutagenesis and sequencing work. pIFDC2 (Xie et al. 2011) is an in-frame deletion (IFD) cassette vector, which uses a highly expressed constitutive promoter to drive the expression of a synthetic operon containing both a positive selection marker (*ermAM*) and a negative selection marker (-pheS*) (Kast 1994). Approximately a 500 base pair (bp) amplification of DNA upstream of *lanA* (primers MutA-UpF and MutA-UpR-IDH) and ˜500 bp amplification of DNA downstream of *lanA* (primers MutA-DnF-erm and MutA-DnR) were generated. These DNA fragments were attached to the 5′ and 3′ end of the IFDC2 cassette, respectively. Transformation of this PCR-amplified product with *S. mutans* JH1140 ATCC 55676 generated the *S. mutans* strain Δ*lanA*/IFDC2 (Table 1). *S. mutans* natural competence pathway was used for transforming PCR and plasmid products. Natural competence can be activated using a competent-stimulating peptide (CSP) (Li et al. 2002). An overnight culture of *S. mutans* strain Δ*lanA*/IFDC2 was diluted to 0.1 OD_600_ and grown to 0.25 OD_600_ before the addition of 2 *μ*L of 10 *μ*g/mL CSP to 200 *μ*L of bacterial suspension. After 30 min of incubation, 1 *μ*L of the PCR product of the IFDC2 cassette spanned by upstream and downstream DNA from the structural gene (*lanA*) was added to the cells. After 4–5 h of incubation, 50 *μ*L of solution was plated on THyex plates containing 15 *μ*g/mL of erythromycin. Colonies that grew in the presence of erythromycin were sequenced to confirm that the IFDC2 cassette replaced the *lanA* gene. This strain was used for subsequent transformations of plasmids containing leader peptide mutations. Leader peptide mutations were introduced into the *lanA* gene by two-step PCR. The primers used to generate each leader peptide mutation are listed in Table 2. The mutations were then inserted into pCR®2.1-TOPO® vector (Life Technologies, Grand Island, NY) according to the provided protocol. The transformants were sequenced by upstream and downstream primers approximately 300 bp from *lanA* using primers MutAseqF and MutAseqR. *S. mutans* Δ*lanA*/IFDC2 was transformed with the same protocol above with 1 *μ*L of cloned pCR®2.1-TOPO® vector. The transformants of the leader peptide mutants were plated on THyex plates with 4 mg/mL of chloro-phenylalanine. Colonies growing in the presence of chloro-phenylalanine represent the loss of the IFDC2 cassette and the insertion of the *lanA* with the expected mutation. Colonies from these plates were identically spotted on THyex and THyex with erythromycin to remove false positives from the screen. Mutants were further confirmed by sequencing.

**Table 2:** Forward and reverse primers used in the study

Primer	Sequence
MutA-UpF-long	GCT TCA ATT CTT AAA TCT AAT TTG AAT CAG CTT TTA TAA A
MutA-DnR-long	TCG GAT CAC TAT GTA GTA ACT CAA TGG GAT CCA TCG
MutAseq-F	GAG GCT AAT GGT GGT ATT ATATTATTG
MutAseq-R	ACC AAG GAC TTC TAA TAA TTG TG
MutA-UpF	GCT TCA ATT CTT AAA TCT AAT TTG AAT C
MutA-UpR-IDH	GAG TGT TAT TGT TGC TCG GAC GAG TAT CTG GAT CGT C
MutA-DnF-erm	GGT ATA CTA CTG ACA GCT TCT TGT ATA AAA GAT TTA GAT TGT GCC
MutA-DnR	TCG GAT CAC TAT GTA ACT CAA
N-6xHis-F	CAT CAT CAT CAT CAT CAT GAA GTC CTT GGT ACT GAA AC
N-6xHis-R	ATG ATG ATG ATG ATG ATG CAT AAT ATC CTC CTT TTT CAT GTG
N+6xHisF	GGA GGA TAT TAT GCA TCA TCA TCA TCA TCA TTC AAA CAC ACA ATT ATT AG
N+6xHisR	CTA ATA ATT GTG TGT TTG AAT GAT GAT GAT GAT GAT GCA TAA TAT CCT CC
Δ(-40-33)-F	GAA AAA GGA GGA TAT TAT GCT TGG TAC TGA AAC TTT T
Δ(-40-33)-R	AAA AGT TTC AGT ACC AAG CAT AAT ATC CTC CTT TTT C
Δ(-40-28)-F	GAA AAA GGA GGA TAT TAT GTT TGA TGT TCA AGA AGA TC
Δ(-40-28)-R	GAT CTT CTT GAA CAT CAA ACA TAA TAT CCT CCT TTT TC
Δ(-40-24)-F	GAA AAA GGA GGA TAT TAT GGAA GAT CTC TTT GCT
Δ(-40-24)-R	AGC AAA GAG ATC TTC CAT AAT ATC CTC CTT TTT C
Δ(-40-37)-F	GAA AAA GGA GGA TAT TAT GTT ATT AGA AGT CCT TGG T
Δ(-40-37)-R	ACC AAG GAC TTC TAA TAA CAT AAT ATC CTC CTT TTT C
Δ(-36-33)-F	TAT TAT GTC AAA CAC ACA ACT TGG TAC TGA AAC TTT T
Δ(-36-33)-R	AAA AGT TTC AGT ACC AAG TTG TGT GTT TGA CAT AAT A
Δ(-32-28)-F	CAC ACA ATT ATT AGA AGT CTT TGA TGT TCA AGA AGA TC
Δ(-32-28)-R	GAT CTT CTT GAA CAT CAA AGA CTT CTA ATA ATT GTG TG
Δ(-27-24)-F	CTT GGT ACT GAA ACT GAA GAT CTC TTT GCT
Δ(-27-24)-R	AGC AAA GAG ATC TTC AGT TTC AGT ACC AAG
Δ(-23-20)-F	GAA ACT TTT GAT GTT CAA GCT TTT GAT ACA ACA GTA
Δ(-23-20)-R	TAC TGT TGT ATC AAA AGC TTG AAC ATC AAA AGT TTC
F(-27)A-F	ACT GAA ACT GCT GAT GTT CAA G
F(-27)A-R	CTT GAA CAT CAG CAG TTT CAG T
D(-26)A-F	TGA AAC TTT TGC TGT TCA AGA AG
D(-26)A-R	CTT CTT GAA CAG CAA AAG TTT CA
V(-25)A-F	AAC TTT TGA TGC TCA AGA AGA TC
V(-25)A-R	GAT CTT CTT GAG CAT CAA AAG TT
Q(-24)A-F	TTT GAT GTT GCA GAA GAT CTC T
Q(-24)A-R	AGA GAT CTT CTG CAA CAT CAA A
E(-23)A-F	GTC CTT GGT ACT GAA GCT TTT GAT GTT CAA GAA
E(-23)A-R	TTC TTG AAC ATC AAA AGC TTC AGT ACC AAG GAC
D(-22)A-F	TTT GAT GTT CAA GAA GCT CTC TTT GCT TTT GAT
D(-22)A-R	ATC AAA AGC AAA GAG AGC TTC TTG AAC ATC AAA
L(-21)A-F	GAT GTT CAA GAA GAT GCC TTT GCT TTT GAT ACA
L(-21)A-R	TGT ATC AAA AGC AAA GGC ATC TTC TTG AAC ATC
F(-20)A-F	GTT CAA GAA GAT CTC GCT GCT TTT GAT ACA ACA
F(-20)A-R	TGT TGT ATC AAA AGC AGC GAG ATC TTC TTG AAC
Q(-24)InsAla-F	TTG ATG TTC AAG CTG AAG ATC TCT
Q(-24)InsAla-R	AGA GAT CTT CAG CTT GAA CAT CAA
E(-23)InsAla-F	GTT CAA GAA GCA GAT CTC TTT G
E(-23)InsAla-R	CAA AGA GAT CTG CTT CTT GAA C
D(-22)InsAla-F	GAT GTT CAA GAA GAT GTC CTC TTT GCT TTT GAT
D(-22)InsAla-R	ATC AAA AGC AAA GAG GAC ATC TTC TTG AAC ATC
L(-21)InsAla-F	GAA GAT CTC GCA TTT GCT TTT G
L(-21)InsAla-R	CAA AAG CAA ATG CGA GAT CTT C
Δ(-21)A-F	TGT TGT ATC AAA AGC GAG ATC TTC TTG AAC
Δ(-21)A-R	GTT CAA GAA GAT CTC GCT TTT GAT ACA ACA
Δ(-20)A-F	TGT TGT ATC AAA AGC GAG ATC TTC TTG AAC
Δ(-20)A-R	GTT CAA GAA GAT CTC GCT TTT GAT ACA ACA
Δ(-19)A-F	TTC AAG AAG ATC TCT TTT TTG ATA CAA CAG ATA C
Δ(-19)A-R	GTA TCT GTT GTA TCA AAA AAG AGA TCT TCT TGA A
AALF-F	ACT TTT GAT GTT CAA GCA GCT CTC TTT GCT TTT GAT
AALF-R	ATC AAA AGC AAA GAG AGC TGC TTG AAC ATC AAA AGT
EDED-F	GAT GTT CAA GAA GAT GAG GAT GCT TTT GAT ACA ACA
EDED-R	TGT TGT ATC AAA AGC ATC CTC ATC TTC TTG AAC ATC

### Deferred antagonism assay

The deferred antagonism assay was performed as previously reported (Chen et al. 2013). *S. mutans* wild-type and mutant strains were grown overnight in liquid THyex culture. The next morning, the culture was diluted to 0.1 OD_600_ and allowed to grow to a mid-logarithmic phase. The culture was then diluted to 0.05 OD_600_ before spotting 2 *μ*l of the bacterial suspension on fresh prewarmed THyex plates. A duplicate of triplicate spots were tested for each strain with wild type and Δ*lanA* serving as positive and negative controls, respectively. The plates were incubated 18 h at 37°C in a candle jar. The next day the bacterial colonies were heat killed at 65°C for one and a half hours and then cooled to 37°C. *M. luteus* from a fresh overnight plate was used to inoculate prewarmed THyex broth and grown at 37°C to a mid-logarithmic phase. The culture was then diluted to 0.2 OD_600_ and diluted 25-fold in prewarmed (42°C) top agar. 5 mL of top agar containing the bacterial suspension was then poured onto each heat-killed plate and incubated overnight at 37°C. The area for each zone of inhibition was calculated and compared to wild-type zones of inhibition. The activity of the purified variants were determined using the same conditions for overlaying the indicator strain, in which 5 *μ*L of the extracted variants were spotted on the prewarmed THyex plates after being overlayed with *M. luteus*. Student *t*-test was the statistical method used to determine significance (*P* < 0.05).

### Isolation of mutacin 1140 leader peptide variants

Mutacin 1140 and variants of mutacin 1140 were isolated as reported previously (Qi et al. 1999). A modified THyex media was used as the fermentation media for inoculation. The media contained 30 g/L Todd Hewitt, 3 g/L yeast extract, 1 g/L NaH_2_PO_4_, 0.2 g/L Na_2_HPO_4_, 0.7 g/L MgSO_4_, 0.005 g/L FeSO_4_, 0.005 g/L MnSO_4_, and 0.3% agar. A total of 500 mL of the semi-solid fermentation media was placed in a 1 L Erlenmayer flask and stab inoculated using an inoculating needle. The inoculum was placed at 37°C for 72 h, and immediately frozen at −80°C. The media was then thawed in a 55°C water bath for 1 h. The inoculum was then placed in 250 mL centrifuge bottles and centrifuged at 20,000 g for 30 min. The collected supernatant was pooled, mixed with chloroform at a 1:1 ratio, and shaken vigorously. The mixture was centrifuged again at 20,000 g for 30 min. The phase between the aqueous and chloroform layers was collected and allowed to dry overnight. The dried precipitate was resuspended in 35% acetonitrile and tested by deferred antagonism assay for activity. The crude extract was run on either a semi-prep C18 column (Agilent® ZORBAX, (Agilent Technologies, Santa Clara, CA) ODS, C18, 5 *μ*m, 4.6 × 250 mm) or analytical column as reported previously (Chen et al. 2013). CDAP (1-cyano-4-dimethylaminopyridinium tetrafluoroborate) and Tris [2- carboxyethyl] phosphine (TCEP) was used to determine whether the isolated products had any free thiols resulting from a lack of cyclase activity (Pipes et al. 2005). We followed the procedure as has been previously reported by Kluskens et al. (Kluskens et al. 2005) with slight modification. We used 0.1 N hydrochloric acid to dissolve CDAP. As a positive control, we used an extended analog of the chemotactic peptide resact (LRGGGVCGPAGTVCGYGGG-NH2) (Singh et al. 1988). The purified products were confirmed by mass on a Shimadzu® MALDI-MS (Shimadzu, Kyoto, Japan) on both linear and reflectron modes.

## Results

### Size matters

**Figure 2:**
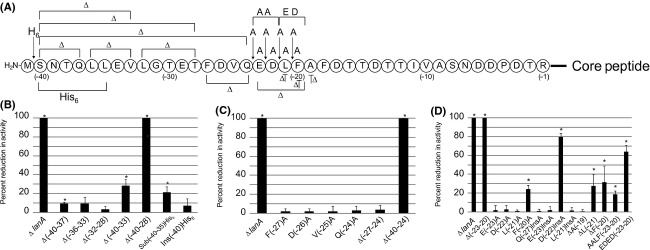
Identification of structural elements within the mutacin 1140 leader peptide that are important for bioactivity. (A) Covalent structure representation of the mutations made on the leader peptide. Bioactivity for leader peptide mutants were measured as the percent difference in the zone of inhibition between wild-type and the mutant strains. Δ*lanA* strain was used as a negative control for bioactivity in all experiments. The change in activity was measured for: (B) N-terminal deletions of the leader peptide, (C) mutations in the proposed FNLD-type box, (D) mutation in a new box, For each mutation, the bioactivity has been compared to the activity of wild-type *S. mutans* JH1140 strain. Statistical method used was Student *t*-test and the asterisk signifies statistical significance (*P* < 0.05).

**Figure 3:**
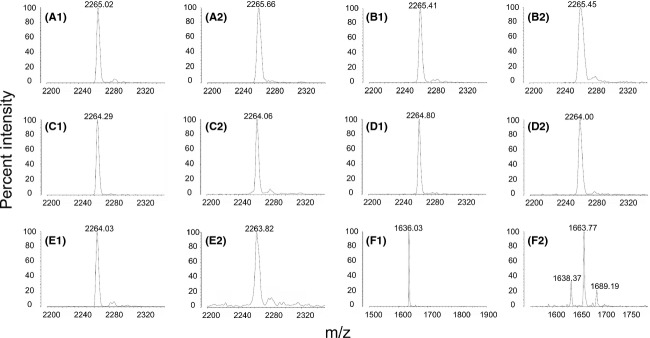
Cyanylation of free thiols by CDAP. (A) MALDI-TOF MS of peptide from Δ(-40-33) strain (A1) and CDAP-treated peptide (A2); (B) MALDI-TOF MS of peptide from AALF strain (B1) and CDAP-treated peptide (B2); (C) MALDI-TOF MS of peptide from ΔL-20 strain (C1) and CDAP-treated peptide (C2); (D) MALDI-TOF MS of peptide from ΔF-19 (D1) and CDAP-treated peptide (D2); (E) MALDI-TOF MS of peptide from F-19A (E1) and CDAP-treated peptide (E2); (F) MALDI-TOF MS of positive control peptide resact (F1) and CDAP-treated peptide (F2). None of the isolated peptides from *S. mutans*-mutant strains reacted with CDAP, while the positive control was cyanylated.

The mutacin 1140 leader peptide (Fig. 1B) is 18 and 11 amino acids longer than nisin A and epidermin leader peptides, respectively. Small consecutive truncations of four and five amino acids were made starting at the (-40) position corresponding to the amino acid after N-terminal methionine (Fig. 2A). Deletion mutations between residues Δ(-40 to -37), Δ(-36 to -33), Δ(-32 to -28) were investigated to determine whether there are regions of structural importance in the extended leader peptide (Fig. 2A). These mutations had little impact on the bioactivity of the mutant strains as determined by deferred antagonism assays (Fig. 2B). This assay is a sensitive quantitative measurement of bioactivity for mutacin 1140 production (Chen et al. 2013). Each strain of *S. mutans* is grown under identical conditions and the bioactivity is assessed by calculating the percent differences in the area of the zone of inhibition between mutant strains and wild-type strain. Reductions in activity suggest that less of mutacin 1140 is made or the biosynthesis of mutacin 1140 by the bacterium is altered leading to the synthesis of less active products. Progressively longer truncations from residues Δ(-40 to -33) and Δ(-40 to -28) were subsequently measured for bioactivity (Fig. 2A and B). The deletion mutants were reduced in bioactivity and the loss in bioactivity increased with the length of the deletion. The progressive loss in bioactivity with increasing size of deletion suggests that the length of the leader peptide is important for the biosynthesis of mutacin 1140. Mutacin 1140 was isolated from the Δ(-40 to -33) deletion mutant and its mass was 2265 Da, as predicted for a core peptide that has successfully undergone all dehydrations and decarboxylation (Table 3). There was no cyanylation of free thiols by CDAP, suggesting that all lanthionine rings were formed by the cyclase (Fig. 3). The substitution of six residues (-40 to -35) with histidines resulted in a statistically significant decrease in bioactivity, whereas, an insertion of six histidines between residues (-41 and -40) position resulted in no significant loss in bioactivity (Fig. 2B). These results further emphasize the importance of the leader peptide's predicted secondary structure for bioactivity (Fig. 1C). Secondary structure analysis using SOPMA (Geourjon and Deleage 1995) predicts that the N-terminal end of the leader peptide is an alpha helix, while the C-terminal end is a random coil (Fig. 1C). The insertion of six histidines at the N-terminal end did not affect bioactivity because they presumably extend outside of a binding cleft, whereas the substitution of six histidines is within the leader peptide-binding cleft of a PTM enzyme. The substitution of six histidines within this region is predicted by SOPMA to change the secondary structure to a random coil. Furthermore, the substitution of six histidines within this region would contribute to steric interference of binding. These results suggest that length of the first half of the leader peptide sequence, and possibly secondary structure, is more important for the biosynthesis of mutacin 1140 than the actual amino acid sequence. The lack of sequence specificity for the leader peptide has also been reported in other lantibiotic systems (van der Meer et al. 1994; Neis et al. 1997; Plat et al. 2011, 2013).

**Table 3:** MALDI-MS data for isolated mutacin 1140 products from *Streptococcus mutans* JH1140

Strain	Mass (Da)
*S. mutans* ATCC 55676	2264.63 ± 1
*S. mutans* Δ(-40-33)	2264.63 ± 1
*S. mutans* Δ(-40-28)	ND
*S. mutans* Δ(-40-24)	ND
*S. mutans* Δ(-40-37)	2264.63 ± 1
*S. mutans* Δ(-36-33)	2264.63 ± 1
*S. mutans* Δ(-32-28)	2264.63 ± 1
*S. mutans* Δ(-27-24)	2264.63 ± 1
*S. mutans* Δ(-23-20)	ND
*S. mutans* F(-27)A	2264.63 ± 1
*S. mutans* D(-26)A	2264.63 ± 1
*S. mutans* V(-25)A	2264.63 ± 1
*S. mutans* Q(-24)A	2264.63 ± 1
*S. mutans* Q(-24)insA	2264.63 ± 1
*S. mutans* E(-23)A	2264.63 ± 1
*S. mutans* E(-23)insA	2264.63 ± 1
*S. mutans* D(-22)A	2264.63 ± 1
*S. mutans* D(-22)insA	ND
*S. mutans* L(-21)A	2264.63 ± 1
*S. mutans* Δ(-21)	2264.63 ± 1
*S. mutans* L(-21)insA	2264.63 ± 1
*S. mutans* F(-20)A	2264.63 ± 1
*S. mutans* Δ(-20)	2264.63 ± 1
*S. mutans* Δ(-19)	2264.63 ± 1
*S. mutans* AALF	2264.63 ± 1
*S. mutans* EDED	ND
*S. mutans* N+6xhis	2264.63 ± 1
*S. mutans* N6xhis	2264.63 ± 1

### FNLD box

A comparison of leader peptide sequences of class I lantibiotics shows a conserved sequence within the first half of the leader peptide (Fig. 1B). The F(N/D)LD box appears to be highly conserved among different lantibiotics (Oman and van der Donk 2010; Plat et al. 2013), but the predicted box sequence for mutacin 1140 is different compared to other lantibiotics. The box for mutacin 1140 was predicted to have the sequence FDVQ located at the (-27 to -24) region of the leader peptide (Chatterjee et al. 2005). We individually substituted the amino acids FDVQ with alanine to determine their importance for bioactivity (Fig. 2A and C). Alanine substitutions did not exhibit any change in activity. Given the possibility that the effect of single point mutations could be masked by the presence of other amino acids within the sequence, we deleted the whole FDVQ box Δ(-27 to -24) from the leader peptide. The truncation also did not significantly reduce the bioactivity (Fig. 2A and C). We isolated the lantibiotic from the culture and determined the mass. The mass of the mutant product in combination with its bioactivity suggested that the core peptide underwent all post translational modifications (Table 3). These observations are incompatible with what has been reported for other related lantibiotics. The substitution at the F(-18) position of nisin with an alanine was shown to reduce the bioactivity of this strain and the deletion of the F(N/D)LD box of nisin abolished production of the lantibiotic (Plat et al. 2011). We therefore searched downstream for a new box.

### A new box

Considering how the deletion of the predicted motif for mutacin 1140 did not affect production, additional deletion mutants were made to determine whether or not mutacin 1140 leader peptide has a unique “box motif” as has been characterized for nisin (Kluskens et al. 2005; Mavaro et al. 2011; Plat et al. 2011; Abts et al. 2013). A deletion of four residues Δ(-23 to -20), positioned at the C-terminal end of the predicted FDVQ box described above, resulted in a complete loss in bioactivity (Fig. 2A and D). The box has an “EDLF” motif, which is a different primary sequence from other related lantibiotics. The loss of activity may be attributed to a loss in leader peptide affinity to the binding cleft of a PTM enzyme (Kluskens et al. 2005; Mavaro et al. 2011; Plat et al. 2011; Abts et al. 2013). However, the deletion of 17 amino acids Δ(-40 to -24) resulted in nearly a complete loss in bioactivity, suggesting that this region is also capable of stabilizing an interaction within the binding cleft of a PTM enzyme. It would be unusual for the loss of just four amino acids Δ(-23 to -20) to result in a complete loss of activity, unless this region is important for anchoring the leader peptide at the correct position in the binding cleft or is essential for activating a functional conformation of one of the PTM enzymes. To test the importance of site-specific amino acids that are necessary for binding, we substituted the EDLF box with alanine residues. Each of these alanine substitutions for E(-23)A, D(-22)A, and L(-21)A resulted in no loss in bioactivity (Fig. 2A and D). The alanine substitution for F(-20)A resulted in approximately a 24% reduction in bioactivity. An insertion of alanine residues in the region of the putative EDLF box was evaluated. The Q(-24)InsAla and E(-23)InsAla mutants did not disrupt the bioactivity. The D(-22)InsAla mutant resulted in an 88% reduction in activity, while the L(-21)InsAla did not result in any significant loss in bioactivity. Furthermore, a deletion at A(-19) position did not result in any reduction in bioactivity. Yet, a deletion at L(-21) or F(-20) resulted in approximately 25% reduction in activity. These mutations point to the importance of all four of the EDLF amino acids in the box. For example, EDLF, EADLF, EDLAF, DLF, and EDF still have normal bioactivity, while EDLA, EDF, and EDALF had a significant reduction in bioactivity (Fig. 2A and D). The relative position of the acidic amino acids (ED) from the hydrophobic amino acids (LF) appears to be important for bioactivity. Each of the acidic and hydrophobic amino acids is able to compensate for the substitution of a single alanine residue in the sequence without resulting in a large loss in bioactivity. To test the requirement of an acidic amino acid relative to the position of hydrophobic amino acid, the mutants AALF and EDED boxes were tested. Each of these mutations resulted in a significant loss in bioactivity (Fig. 2A and D). AALF activity was reduced by ˜25% and EDED activity was reduced by ˜65%. These mutations further indicate the importance of the EDLF box, specifically the latter two amino acids in the motif. This motif could be a potential binding site for the dehydratase LanB. We were unable to isolate any product from the culture liquor in the EDED-mutant strain, which suggests that the motif is important for biosynthesis. We were able to isolate the AALF-mutant product which had a mass of 2265 Da. Furthermore, all of the substitution or insertion mutations within this region resulted in a product with a mass of 2265 Da, which is the mass of the product that has undergone all dehydration and decarboxylation modifications (Table 3). There was no cyanylation of free thiols by CDAP in the AALF, ΔL-20, ΔF-19, and F-19A box mutants, suggesting that all lanthionine rings were formed by the cyclase (Fig. 3). The reduction in bioactivity in connection with the isolation of only fully modified mutacin 1140 supports the notion that core peptide PTM enzyme modifications are important for transport.

## Discussion

Due to their unique structure, lack of resistance, and wide array of activity, lantibiotics have become a prime candidate for development of new therapeutics. Spontaneous resistance to lantibiotics like mutacin 1140 is highly unlikely given their mechanism of action (Breukink et al. 1999; Hasper et al. 2004, 2006; Smith et al. 2008). Mutacin 1140, a lantibiotic produced by *S. mutans* JH1140, has been shown to be active against serious pathogens like MRSA (Ghobrial et al. 2009). In this study, we investigated the role of structural regions within the leader peptide and core peptide for biosynthesis. Mutacin 1140 leader peptide sequence is different and longer than other lantibiotic leader peptides, while the core peptide sequence is similar to epidermin and nisin (Fig. 1). A better understanding of the role of the leader peptide in the post translational modification of lantibiotics will allow us to advance the use of the PTM enzymes for the synthesis of mutacin 1140, novel lantibiotics, and therapeutic peptides.

In nisin, N-terminal mutagenesis studies upstream of the conserved FNLD box were tolerated by the PTM enzymes (Blaesse et al. 2000). We predicted that N-terminal truncations of the mutacin 1140 leader peptide would also be well tolerated and that the additional length may not be important for bioactivity. However, we did see a loss in activity with the increasing length of the deletion upstream of the newly identified box. Given that the small truncations of four and five amino acids over the same regions as covered by the larger deletion mutants did not reduce the bioactivity, site-specific amino acids within this region of the leader peptide may not be important for recognition by the PTM enzymes. Presumably, hydrogen bonds are formed between the peptide backbone of the N-terminal portion of the leader peptide and PTM enzyme. This type of interaction and not the interaction of the amino acid side chains of the leader peptide would account for the lack of sequence specificity. Other studies support a helical structure for the leader peptide in the binding cleft of the dehydratase of lacticin 481 and that secondary structure is important for activity (Patton et al. 2008; Oman and van der Donk 2010). These studies have shown that insertion of prolines, which would disrupt the helical structure, would reduce the bioactivity (Patton et al. 2008; Oman and van der Donk 2010). Secondary structure prediction suggests that the N-terminal portion of mutacin 1140 leader peptide is helical, while the C-terminal end is a random coil. The leader peptide sequence within the N-terminal region is presumably reserved to a subset of amino acids that will not disrupt the secondary structure or is reserved to amino acids that will not contribute to the steric hindrance of binding. Plat et al. have also reported a lack of sequence specificity within the nisin leader peptide (Plat et al. 2011).

Some models for the binding cleft suggest that both LanB and LanC have their own distinct regions for binding the leader peptide or that the interaction of LanB and LanC form the binding cleft for the leader peptide (Plat et al. 2013). There are two publications pertaining to the interaction of LanB and LanC that appear to have conflicting results. Mavaro et al. showed a 1:1 stoichiometry of binding of prenisin, dehydrated nisin, and fully modified nisin to LanB. The affinity of the prenisin with leader peptide was approximately 1 *μ*mol/L and the affinity of fully modified nisin with leader peptide was a log higher. The dehydrated form had a twofold higher affinity to LanB than prenisin. These data would suggest that prenisin would prefer to associate with LanB and that cyclization may occur while being bound to LanB. Abts et al. reported the binding of leader peptide to the cyclase LanC (Abts et al. 2013). The prenisin, dehydrated and fully modified nisin had similar binding affinities at approximately 2 *μ*mol/L. In this study, they showed that AALD and FNAA box mutations resulted in a complete loss of binding to LanC. However, synthesis of fully modified nisin was shown by Plat et al., while having similar mutations (Plat et al. 2011). This suggests that cyclase activity is occurring while leader peptide is bound to LanB and that LanC binding is not essential for cyclase activity. One would expect the formation of only a dehydrated nisin and not a biologically active peptide, if leader peptide binding to LanC was required. The study by Abts et al. does clearly show the requirement of the FNLD box for binding to LanC and seems to conflict with the study by Plat et al. (Plat et al. 2011; Abts et al. 2013). In view of these results, it is possible that there are two functional leader peptide-binding sites that are not competing for the leader peptide. Both sites may coordinate the binding of the leader peptide, so that the core peptide can access the catalytic sites of the PTM enzymes. Additional studies will need to be done to discern the importance of these two sites in nisin biosynthesis. Biologically active product was observed in the mutacin 1140 biosynthesis system in the EDLF box mutants AALF and EDED. The AALF mutant had a single product with the mass of a fully modified core peptide, while the peptide in the EDED mutant could not be isolated for mass characterization. The lack of CDAP derivatized products support complete lanthionine ring formation in the AALF-mutant product. Mutations within mutacin 1140 leader peptide box motif have similar characteristics to the mutants reported in the FNDL box in the nisin biosynthesis system.

To date, the biosynthesis of only a handful of lantibiotics has been studied. Additional studies within other lantibiotic biosynthesis systems will promote our understanding of this dynamic process. One important goal to lantibiotic research is the application of the PTM enzymes toward the synthesis of novel therapeutic compounds. Surely, an expanded understanding of PTM enzymes in other lantibiotic systems will promote this endeavor. We believe that the EDLF box is a structurally important region that facilitates alignment of the leader peptide within the binding cleft of the dehydratase LanB, similar to the function of the FNLD box of nisin (Chatterjee et al. 2005; Lubelski et al. 2008; Oman and van der Donk 2010). However, the difference in leader peptide box motif and length suggest that the leader peptide-binding site is evolutionarily unique to other class I lantibiotics. This brings to question, whether lantibiotics should be classified by structural elements within the leader peptide as has been previously reported (Oman and van der Donk 2010) or should they be classified based on core peptide structure. The classification of lantibiotics by leader peptide does suggest evolutionary relatedness in their PTM systems and substrate specificities. Given the complexity of lanthipeptide biosynthesis, classification by leader peptide appears to be a more logical approach. Additional studies aimed at understanding substrate specificity of the PTM enzymes between lantibiotic systems may promote a better understanding of the evolutionary differences between the class I PTM enzymes. Our leader peptide mutagenesis study and the concomitant isolation of the mutagenic products provide a better understanding of mutacin 1140 leader peptide. Furthermore, the studies show that the leader peptide of mutacin 1140 has evolved novel structural elements that promote its interaction with its PTM enzymes and that these structural elements are unique when compared to related class I lantibiotics. Further studies within mutacin 1140 biosynthetic pathway are underway to determine substrate specificity of mutacin 1140 biosynthesis system.
